# Effects of Community-Based Caring Contact in Reducing Thwarted Belongingness Among Postdischarge Young Adults With Self-Harm: Randomized Controlled Trial

**DOI:** 10.2196/43526

**Published:** 2023-08-16

**Authors:** Yik Wa Law, Rita Hui Ting Lok, Byron Chiang, Carmen Chui Shan Lai, Sik Hon Matthew Tsui, Pui Yin Joseph Chung, Siu Chung Leung

**Affiliations:** 1 Department of Social Work and Social Administration University of Hong Kong Hong Kong Hong Kong; 2 The Hong Kong Jockey Club Centre for Suicide Research and Prevention University of Hong Kong Hong Kong Hong Kong; 3 Accident and Emergency Department Queen Mary Hospital Hong Kong Hong Kong; 4 Department of Psychiatry Pamela Youde Nethersole Eastern Hospital Hong Kong Hong Kong; 5 Department of Emergency Medicine School of Clinical Medicine University of Hong Kong Hong Kong Hong Kong

**Keywords:** self-harm, suicidal ideation, volunteers, mobile app, thwarted belongingness, suicide, youth, community, support, treatment, effectiveness, risk, patient, intervention, model, care, hospital

## Abstract

**Background:**

For patients with self-harm behaviors, the urge to hurt themselves persists after hospital discharge, leading to costly readmissions and even death. Hence, postdischarge intervention programs that reduce self-harm behavior among patients should be part of a cogent community mental health care policy.

**Objective:**

We aimed to determine whether a combination of a self-help mobile app and volunteer support could complement treatment as usual (TAU) to reduce the risk of suicide among these patients.

**Methods:**

We conducted a pragmatic randomized controlled trial on discharged patients aged between 18 and 45 years with self-harm episodes/suicide attempts, all of whom were recruited from 4 hospital emergency departments in Hong Kong. Participants were randomly assigned to one of three groups: (1) mobile app + TAU (“apps”), (2) mobile app + volunteer support + TAU (“volunteers”), or (3) TAU only as the control group (“TAU”). They were asked to submit a mobile app–based questionnaire during 4 measurement time points at monthly intervals.

**Results:**

A total of 40 participants were recruited. Blending volunteer care with a preprogrammed mobile app was found to be effective in improving service compliance. Drawing upon the interpersonal-psychological theory of suicide, our findings suggested that a reduction in perceived burdensomeness and thwarted belongingness through community-based caring contact are linked to improvement in hopelessness, albeit a transient one, and suicide risk.

**Conclusions:**

A combination of volunteer care with a self-help mobile app as a strategy for strengthening the continuity of care can be cautiously implemented for discharged patients at risk of self-harm during the transition from the hospital to a community setting.

**Trial Registration:**

ClinicalTrials.gov NCT03081078; https://clinicaltrials.gov/study/NCT03081078

## Introduction

### Background

Among the 127,801 self-harm records identified from January 1, 2002, to December 31, 2016, in the inpatient units of Hong Kong emergency departments, an alarming 22.45% were readmissions [1]. The greatest risk was observed within the same year of the index self-harm episode, regardless of gender or age [1]. These findings suggest that postdischarge patients with self-harm behaviors may require additional community-based interventions to complement usual care. Clinical trials have shown follow-up contact with such patients significantly reduces self-harm repetition. Some studies have also examined the mechanism by which caring contact as a form of engagement potentially leads to a reduction in suicidal behavior [2-4], but the evidence remains inconclusive [5-7].

The interpersonal-psychological theory of suicide (IPTS) provides a theoretical explanation, positing that social connectedness and assurance buffer against perceived loneliness and burdensomeness, which are closely related to hopelessness and suicidal behaviors [8-11]. It suggests that low thwarted belongingness (TB, “I am alone”) and perceived burdensomeness (PB, “I am a burden”) induce suicide [10].

There is an intrinsic value in ascertaining a community-based intervention that is based on the 3 IPTS-related concepts in reducing suicide risks among discharged individuals, especially when it has never been done before. In particular, we hypothesized that community-based interventions that increase TB and PB will reduce suicide risk among patients who receive only treatment as usual (TAU). TAU was prescribed by the hospitals as per individual patients’ medical conditions, which may include psychiatric outpatient follow-up, medication, and psychosocial interventions related to the index self-harm episode.

In this study, 2 community-based interventions were tested. One intervention relied on mobile apps to deliver programmed care for mental health care purposes. Previous studies have examined the efficacy of mobile apps on mental illness management and the prevention of behavioral problems, as well as their use as interventions aimed at reducing depression, anxiety, and substance use in randomized controlled trials (RCTs) [12]. Although the results are mixed, they can still be used to facilitate evidence-based self-monitoring [13]. The second intervention combines programmed care on a mobile app and personalized care delivered by trained volunteers. Volunteers are vital in the suicide prevention pipeline, fielding crisis calls and providing emotional support to callers from around the world since the 1950s [14-17], and they continued to feature prominently during the COVID-19 pandemic [18]. Unlike boilerplate responses from mobile apps, the human element in personalized care is better received by people in emotional distress, which could be key to reducing levels of hopelessness among individuals who practice or are at risk of self-harm. However, owing to several methodological issues, particularly a lack of rigorous hypothesis testing research, the effects of volunteer care or peer support in suicide prevention have yet to be conclusively defined [16,19].

### Aims

This study aimed to determine the efficacy of complementary community-based caring contact via a mobile app regardless of volunteer support to TAU (psychiatric and psychosocial treatments) in reducing the suicide risk among postdischarge young adults practicing self-harm. This study also aimed to suggest tools supported by empirical evidence to engage and support this high-risk group who often default on prescribed treatments. The anticipated effects were hypothesized as a reduction in interpersonal relationship-state TB and PB, hopelessness, and suicide risk in patients aged between 18 and 45 years with self-harm episodes or suicide attempts discharged from the emergency departments of 4 local hospitals. They were also expected to exhibit an improvement in self-reported compliance with treatment.

## Methods

### Overview

This study took the form of a pragmatic RCT examining the effects of community-based caring contact via a mobile app regardless of volunteer support relative to TAU on young adults with self-harm behaviors. After providing informed written consent for participation, participants were randomly assigned to 1 of 3 groups for a 3-month observation period: (1) mobile app + TAU (“apps”), (2) mobile app + volunteer support + TAU (“volunteers”), or (3) TAU only as the control group (“TAU”). The intervention period spanned between T0 and T2. Group assignments were based on a pregenerated sequence returned by the pseudorandom generator. Each participant was asked to complete a questionnaire at each of the 4 measurement time points (T0 = baseline; T1 = 1 month; T2 = 2 months; T3 = 3 months after T0 (post intervention). All participants completed the baseline questionnaire during a face-to-face interview with a research team member around hospital discharge, with the rest self-administered via a mobile app. Incentives were offered for every completed questionnaire.

### Participants

**Figure 1:**
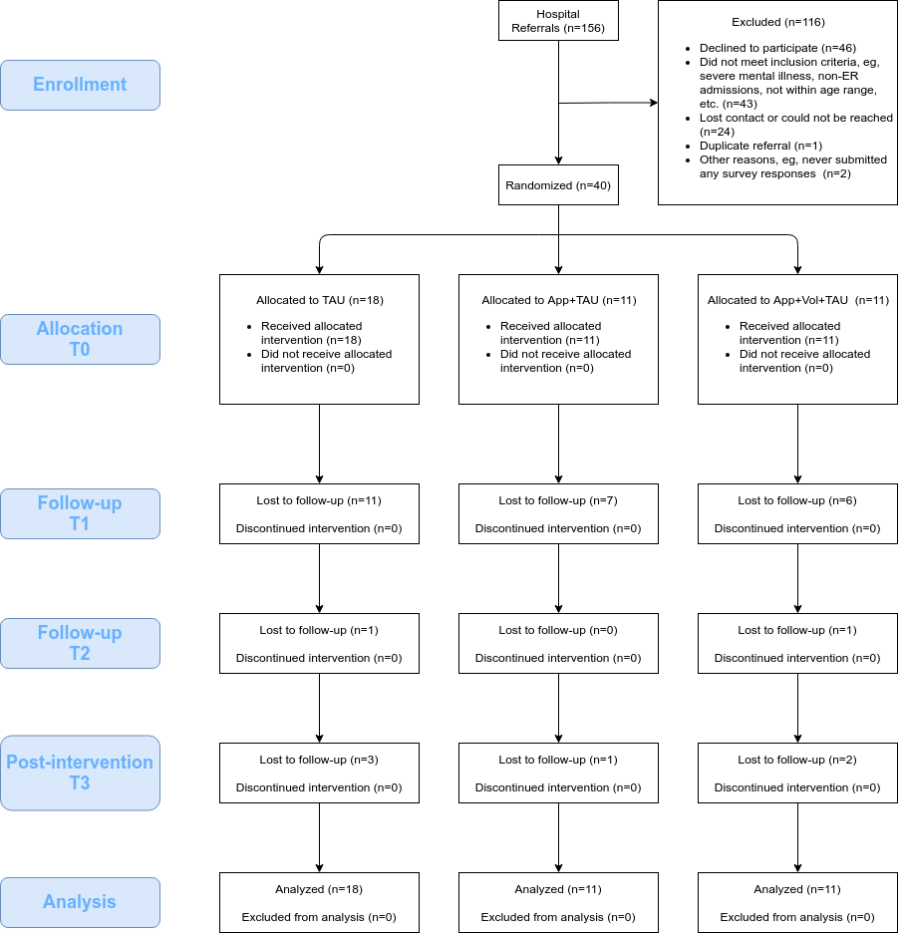
CONSORT (Consolidated Standards of Reporting Trials) flow diagram of research participant recruitment. ER: emergency room; TAU: treatment as usual.

An International Classification of Disease 10th revision (ICD-10)–based definition was used to encompass individuals with an index episode of self-poisoning or self-injury regardless of suicide intent (ie, X60 to X84 and R45.8) [20,21]. Other inclusion criteria were (1) 18 to 45 years of age, (2) admission to the emergency departments of any of the 4 public general hospitals, and (3) approval from a psychiatrist or psychiatric nurse. Young adults were targeted for their digital proficiency and altruism, which makes them less likely to turn down volunteer-initiated contact [22]. The exclusion criteria were (1) any DSM IV-TR (Diagnostic and Statistical Manual of Mental Disorders, 4th Edition, Text Revision) Axis II personality disorder and (2) severe psychotic mental illness or bipolar disorder because patients with these diagnoses may be too demanding for lay volunteers to deal with. Among the 156 referrals given, 1 was a duplicate referral, and 46 potential candidates explicitly turned down the invitation. Another 43 failed to meet the inclusion criteria, and 26 people failed to show up to the study after giving (verbal) consent. The remaining 40 referrals signed a consent to participate in this study. Figure 1 contains a more detailed breakdown of the recruitment process.

### Procedures

Admitted patients were subjected to the standard of care in emergency departments before they went through the vetting process that identified them as potential participants. Patients who met the inclusion and exclusion criteria were notified and briefed on the study by a research team member. Then, they were asked to sign a written informed consent to participate in this study. Immediately upon giving consent, the participants downloaded the app onto their mobile devices and completed a baseline questionnaire. Group assignments and corresponding interventions were given via the app. Grocery gift coupons were offered as incentives for every questionnaire completed thereafter.

### Mobile App Features

The main pages of the mobile app were positive quotes/messages displayed on the home page, a mental health information library, emergency information including the number of a 24-hour suicide prevention hotline, a medical follow-up appointment reminder, a survey, a user feedback form, and, if applicable, volunteer support services (Table 1).

**Table 1:** Components of mobile app and volunteer interventions focusing on reducing TB^a^ and PB^b^.

IPTS^c^ components			TB			PB
Aims of intervention			Reduce sense of loneliness and increase social connectedness			Reduce self-hate and enhance self-assurance
**Programmed**						
	Mobile app	Self-awareness of daily mood states Reduction in loneliness through automatic reminders on participants’ own support network contacts; for example, direct dial function with social network (friends and family, social and health care resources) Appointment reminders for health care/psychosocial services Positive message of the day			Push message: participants’ prewritten positive messages of self-encouragement Knowledge bank on mental health Stress/mood management skills through various practices	
**Personalized**						
	Volunteer support (at least 2 contacts per month made by volunteers for each assigned participant)	Emotional support, active listening, and encouragement to comply with treatment			Provision of positive feedback to and assisting participants in self-assurance through positive messages	
**Personalized additional components as a safety measure**						
	Suicide risk screening through simple questions (ie, ask and refer) report crisis to the research team	N/A^d^		N/A		

^a^PB: perceived burdensomeness.

^b^TB: thwarted belongingness.

^c^IPTS: interpersonal-psychological theory of suicide.

^d^N/A: not applicable.

### Volunteer Support

Volunteers aged 25 years or above with at least a bachelor’s degree were recruited and asked to attend six 3-hour training sessions on basic knowledge of suicide prevention, suicide risk screening skills, empathetic communication skills, and other topics. A similar intervention was examined in a pilot study and found to be effective in reducing depressive symptoms and hopelessness [16]. Volunteers who completed the training and met the assessment criteria were invited to take part in this study. They were paired up with other participants of the “volunteers” group and made first contact within 24 hours. Volunteers’ duties included initiating at least 2 contacts per week with the assigned patients, having at least 1 monthly meetup, conducting suicide risk screening, and sending supportive messages and reminders of follow-up treatment. They were also required to report back on the patients’ progress and attend in-service training and supervision sessions (Table 1). Among the 85 volunteers who applied, 29 (34%) were selected to join the training sessions. Of those, 17 (59%) completed the training and committed to a service contract with a list of duties and promised to comply with ethical practices. A total of 11 (28%) participants were successfully paired with trained volunteers.

### Measurements

The expected outcomes were a reduction in suicidal ideation, hopelessness, TB, and PB total scores and an increase in compliance with professional help. Suicidal ideation was measured by the self-report Chinese version of the 4-item short-form Adult Suicidal Ideation Questionnaire (ASIQ4) adapted from the full-form Adult Suicidal Ideation Questionnaire (ASIQ), which has been demonstrated to exhibit a modest level of sensitivity (64%) and superior specificity (76%), as well as a positive predictive value of 8.4% on nonfatal suicidality and suicide deaths at a cut-off of 1 [23]. Hopelessness was measured by the self-report 4-item short-form Beck Hopelessness Scale (BHS4) [24], which was tested in a local panel survey [25] based on the Chinese version developed by Shek [26]. The BHS4 is highly correlated (0.88) with the long-form Beck Hopelessness Scale (BHS), and its area under the curve (95% CI 0.65-0.75) is as strong as that of the BHS in identifying people with suicidal ideation. The cut-off score of 11 for suicidal ideation provides 65.8% sensitivity and 67.3% specificity [25]. TB and PB were assessed by the Interpersonal Needs Questionnaire (INQ), a 15-item measure of beliefs about whether one’s need to belong is met or unmet and self-perceptions of being a burden to others. Each item is rated on a 7-point Likert scale, with higher scores indicating higher levels of TB and PB [11]. Self-reported compliance with prescribed treatment (frequency) in relation to the index self-harm episode, including psychiatric follow-up care (eg, medication, outpatient follow-up treatment, and community psychiatric nursing service) and psychosocial services (eg, “formal help” provided by clinical psychology service, medical social work service), as well as obtaining “informal help” from families and friends, was measured by a self-developed service utilization checklist. Depressive state was treated as a covariate and measured by the Center for Epidemiological Studies Depression Scale (CESD) [27]. CESD scores measured by 2 factors, an affective and somatic symptom factor and an interpersonal problem factor that patients encountered in the past week, were controlled in growth models. The a priori estimated sample size for each group was 36, with projected retention rates of 80%, 70%, and 60% at T1, T2, and T3, respectively, a 2-sided significance level (α) of 0.05, and a statistical power (1-β) of 0.8 [28]. Participants were assigned to groups by simple random sampling.

### Data Analysis

Chi-square (categorical variables) and Kruskal-Wallis (continuous variables) tests at a significance level of *P*<.05 were performed to detect differences in the distribution of the key variables, (ie, age, sex, hospital, and depressive state) among the 3 groups. Correlations among the measurements were also tested at T0 to verify their association with each other at baseline. Compliance was a binary variable coded in terms of true (1) if the participant reported seeking informal or formal help and false (0) otherwise. An intention-to-treat (ITT) analysis was conducted to compare the participants in all groups, including those who did not comply with the assigned interventions and dropouts, to ensure that all groups remained the same apart from random variation, with the specific purpose of not overestimating the effects of interventions in a pragmatic trial [29]. Following the ITT approach outlined in [30], all cases with only the baseline record (n=21, 52%) available were selectively imputed as if they were in the “TAU” (control) group. Due to the large rates of attrition, group results were analyzed graphically in a manner similar to single-subject research design plots, and the effects were visually inspected. Python (Python Software Foundation) libraries *pandas* (version 1.4.3), *missingpy* (version 0.2.0), and *scikit-learn* (version 0.24.2) were used for all data analyses, and *seaborn* (version 0.11.1) was used to visualize the data.

### Ethics Approval

This study was approved by the Institutional Review Boards of the Hospital Authority Research Ethics Committee across 4 public hospitals: North District Hospital (ref 2016.155-T), Pamela Youde Nethersole Eastern Hospital (ref HKECREC-2017-002), Queen Mary Hospital (ref UW 16-181) and United Christian Hospital (ref KC/KE-16-0027/FR). The study was registered with the US National Institutes of Health Clinical Trials Registry (NCT03081078). Regarding ethical approval, we are also bound to observe and comply with all applicable requirements under the Hospital Authority’s standard operating procedure, the Declaration of Helsinki, and, if applicable, the International Conference on Harmonization of Technical Requirements for Pharmaceuticals for Human Use Guideline for Good Clinical Practice (ICH-GCP).

Incentives were given as a token of appreciation. The maximum value of compensation allotted to each participant was HK $500 (roughly US $64), including an HK $200 (US $25) supermarket coupon for the baseline questionnaire submission and then an HK $100 (US$ 12.50) supermarket coupon for each returned follow-up questionnaire. Data that could be used to identify the participants were anonymized before data analysis.

## Results

### Descriptive Data of All Cases

A total of 40 participants aged between 18 and 45 years with an index episode of self-poisoning or self-injury, regardless of suicide intent, were recruited between 2018 and 2020 from 4 public hospitals. Participants were randomly assigned to 1 of 3 groups: TAU (n=18, 45%), “apps” (n=11, 28%), and “volunteers” (n=11, 28%). Recruitment at the emergency departments was periodically suspended in 2019 owing to social unrest and in 2020 in the wake of the COVID-19 pandemic, which led to a small sample size with a higher number of participants in the TAU group and a high attrition rate between 55% and 63% at T1. The overall attrition rate measured at the end of the intervention at T2 was between 33.3% and 36.4%. At T0, there were no significant differences among the groups in terms of age, sex, depressive symptoms measured by CESD, or attrition rates. Table 2 shows the age, sex, depressive symptoms, and group of all participants at the 4 time points, and Table 3 shows a descriptive summary of outcomes at 4 time points (as per the protocol). The psychometric responses received at all time points had good internal consistency, with Cronbach alpha values above 0.76, similar to the range reported in the prior literature. The correlations between the measurement scales were statistically significant (Table 4), except for that between hopelessness and PB, again confirming earlier findings in the literature. However, hopelessness was correlated with TB and the overall INQ, suggesting that hopelessness exhibited stronger interdependence with TB than PB.

When the results are considered per protocol, for “volunteer” and “TAU” cases, none of the participants reported readmission to the hospital because of self-harm.

**Table 2:** Age, sex, depressive symptoms, and intervention group of all participants at the 4 time points.

Characteristics				Time points						
				T0 (n=40)		T1 (n=16)		T2 (n=14)		T3 (n=8)
**Age (years), n (%)**										
	17-25		18 (45)		9 (56)		8 (57)		5 (63)	
	26-45		22 (55)		7 (46)		6 (43)		3 (38)	
**Sex, n (%)**										
	Female		29 (73)		7 (44)		9 (64)		4 (50)	
	Male		11 (28)		9 (56)		5 (36)		4 (50)	
**Intervention group**										
	Volunteer		11 (28)		5 (31)		4 (29)		2 (25)	
	App		11 (28)		4 (25)		4 (28.6)		3 (38)	
	TAU^a^		18 (45)		7(44)		6 (43)		3 (38)	
CESD^b^ depressive symptoms, mean (SD)				34 (8.3)		29.8 (10.5)		29.3 (11.9)		33.4 (10.3)
**Breakdown of total score by group, mean (SD)**										
	**CESD**									
		Volunteer	37.6 (6.2)		34.6 (6.7)		34.8 (5.4)		37.3 (9.6)	
		App	35.2 (9.7)		34.4 (9.5)		37.3 (10.3)		30.5 (2.1)	
		TAU	31.2 (7.9)		23 (9.6)		20.3 (10.7)		31.3 (15.5)	
	**Age (years), mean (SD)**									
		Volunteer	25.9 (8.1)		26.2 (10.2)		25.7 (9.6)		27.9 (8.6)	
		App	29.7 (8.7)		29 (9.3)		29.9 (8.8)		30.4 (8.8)	
		TAU	29.4 (9)		29.9 (10.3)		30.3 (9.9)		28.5 (8.7)	

^a^TAU: treatment as usual.

^b^CESD: Center for Epidemiological Studies Depression Scale.

**Table 3:** Descriptive summary of outcomes at the 4 time points.

Components				Time points					
				T0		T1		T2	T3
**Total scores, mean (SD)**									
	ASIQ4^a^			12.5 (7.9)	12 (8.1)		10.1 (9.4)		16.1 (9)
	BHS4^b^			14.2 (4.8)	14.8 (5.5)		14.4 (4.7)		14.6 (5.5)
	INQ^c^			60.5 (17.8)	55.8 (19.1)		53.9 (20.2)		57.5 (23.4)
	INQ-PB^d^			21.3 (8.7)	20.3 (11.2)		18.1 (10)		19.6 (10.2)
	INQ-TB^e^			39.2 (11.4)	35.5 (13.5)		35.9 (12.5)		37.9 (16)
**Breakdown of total scores by group, mean (SD)**									
	**ASIQ4**								
		Volunteer		10.7 (7)	14.6 (8.2)		6.8 (3.9)		15 (11.5)
		App		14.5 (8.1)	13.5 (10.2)		17 (11.5)		21.5 (2.1)
		TAU^f^		12.3 (8.5)	9.3 (7)		7.7 (9.4)		13.7 (10.4)
	**BHS4**								
		Volunteer		14.4 (4.5)	16.2 (5.4)		16.3 (3.4)		18 (7.2)
		App		16 (5.1)	16.8 (5.2)		14.3 (4.6)		11.5 (0.7)
		TAU		12.9 (4.6)	12.7 (5.4)		13.2 (5.7)		13.3 (5)
	**INQ**								
		Volunteer		59.5 (17.1	57.8 (20.2)		48.8 (17.5)		65.3 (34.5)
		App		63.6 (23.4)	60.2 (23.7)		60.5 (25.2)		67 (11.3)
		TAU		59.2 (14.9)	52.1 (15.6)		53 (20.9)		43.3 (13.3)
	**INQ-PB**								
		Volunteer		20.5 (8.8)	24.8 (15.1)		18.3 (8.5)		21 (15)
		App		21.9 (12.2)	19.6 (11.2)		21.8 (12)		19.5 (0.7)
		TAU		21.5 (6.3)	17 (7.2)		15.5 (10.5)		18.3 (11.6)
	**INQ-TB**								
		Volunteer		39.1 (9.8)	33 (7.2)		30.5 (11.2)		44.3 (19.6)
		App		41.7 (13.8)	40.6 (17.8)		38.8 (16.7)		47.5 (12)
		TAU		37.7 (11)	35.1 (14.1)		37.5 (11.5)		25 (6.1)
**Participants reporting service compliance by group, n (%)**									
	**Yes**								
		Volunteer		10 (91)	4 (80)		4 (100)		2 (67)
		App		9 (82)	3 (75)		3 (75)		2 (100)
		TAU		15 (83)	2 (29)		2 (33)		1 (33)
**Participants seeking professional consultation by group, n (%)**									
	**Yes**								
		Volunteer		9 (82)	4 (80)		4 (100)		2 (67)
		App		8 (73)	3 (75)		3 (75)		2 (100)
		TAU		13 (72)	2 (29)		2 (33)		1 (33)
**Participants seeking informal help by group, n (%)**									
	**Yes**								
			Volunteer	9 (82)	3 (60)		3 (75)		1 (33)
			App	7 (64)	2 (50)		3 (75)		2 (100)
			TAU	11 (61)	2 (29)		0 (0)		1 (33)

^a^ASIQ4: 4-item short-form Adult Suicidal Ideation Questionnaire.

^b^BHS4: 4-item short-form Beck Hopelessness Scale.

^c^INQ: Interpersonal Needs Questionnaire.

^d^INQ-PB: Interpersonal Needs Questionnaire-Perceived Burdensomeness items.

^e^INQ-TB: Interpersonal Needs Questionnaire-Thwarted Belongingness items.

^f^TAU: treatment as usual.

**Table 4:** Baseline Holm-Bonferroni—corrected Spearman correlations between measurement scales and subscales.

Variable		ASIQ4^a^	BHS4^b^	CESD^c^	PB^d^	TB^e^	INQ-PB + TB^f^
**ASIQ4**							
	*r*	1	0.475^g^	0.546^g^	0.646^g^	0.48^g^	0.686^g^
	*P* value	—^h^	.016	.004	.000	.015	.004
**BHS4**							
	*r*	0.475^g^	1	0.605^g^	0.375	0.567^g^	0.555^g^
	*P* value	.016	—	.001	.086	.003	.004
**CESD**							
	*r*	0.546^g^	0.605^g^	1	0.537^g^	0.549^g^	0.655^g^
	*P* value	.004	.001	—	.005	.004	<.001
**PB**							
	*r*	0.646^g^	0.375	0.537^g^	1	0.496^g^	0.865^g^
	*P* value	<.001	.086	.005	—	.011	<.001
**TB**							
	*r*	0.48^g^	0.567^g^	0.549^g^	0.496^g^	1	0.848^g^
	*P* value	.015	.003	.004	.011	—	<.001
**INQ-PB + TB**							
	*r*	0.686^g^	0.555^g^	0.655^g^	0.865^g^	0.848^g^	1
	*P* value	0.004	0.004	<.001	<.001	<.001	—

^a^ASIQ4: 4-item short-form Adult Suicidal Ideation Questionnaire.

^b^BHS4: 4-item short-form Beck Hopelessness Scale.

^c^CESD: Center for Epidemiological Studies Depression Scale.

^d^PB: perceived burdensomeness.

^e^TB: thwarted belongingness.

^f^INQ: Interpersonal Needs Questionnaire-Perceived Burdensomeness and Thwarted Belongingness items.

^g^The correlation is significant at a significance level of *P=*.05.

^h^—: not applicable.

### Primary Outcomes

**Figure 2:**
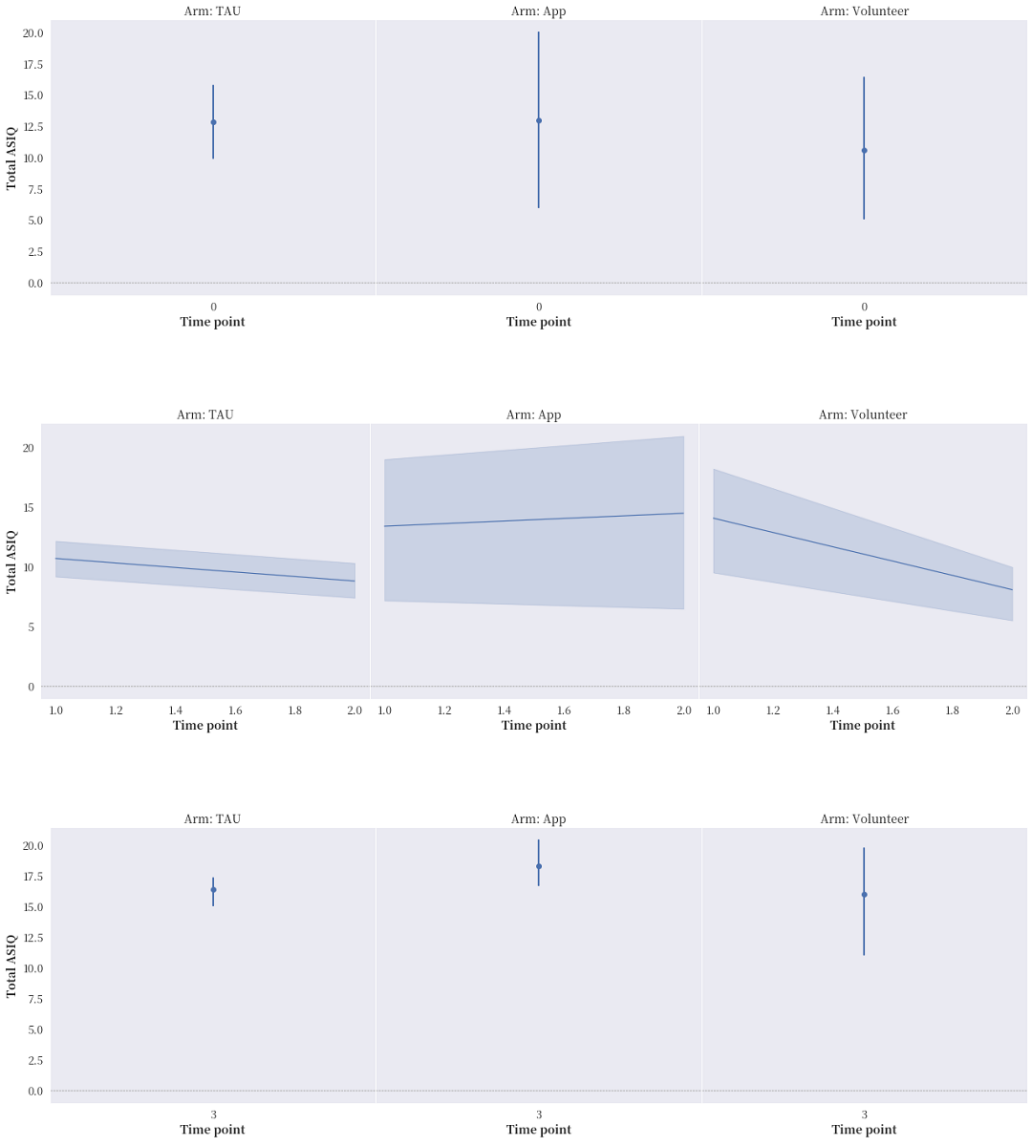
The effects of the intervention on the 4-item short-form Adult Suicidal Ideation Questionnaire (ASIQ4) at the 4 time points, with intention-to-treat (ITT) analysis applied. ASIQ: Adult Suicidal Ideation Questionnaire; TAU: treatment as usual.

**Figure 3:**
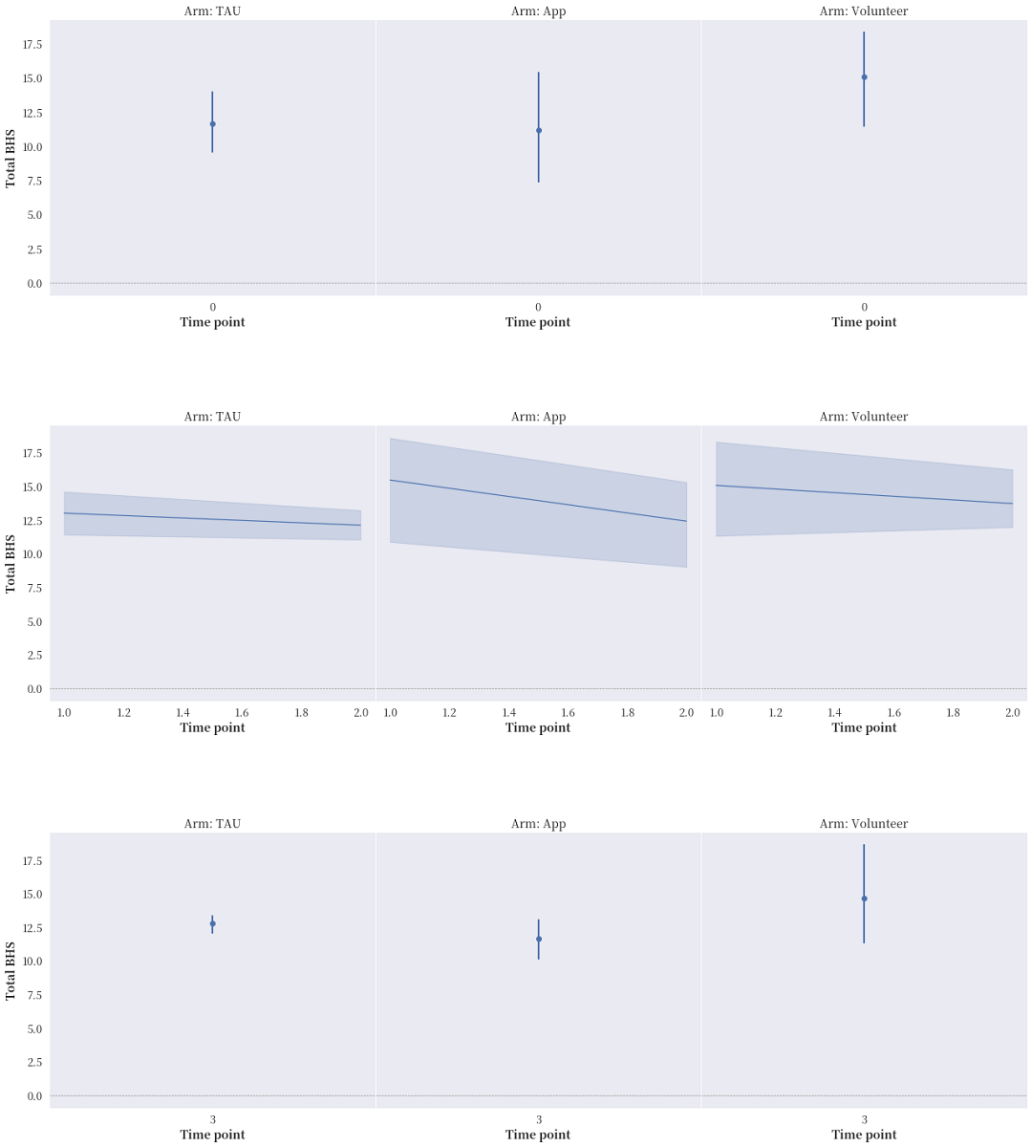
The effects of the intervention on 4-item short-form Beck Hopelessness Scale (BHS4) at the 4 time points, with intention-to-treat (ITT) analysis applied. BHS: Beck Hopelessness Scale; TAU: treatment as usual.

**Figure 4:**
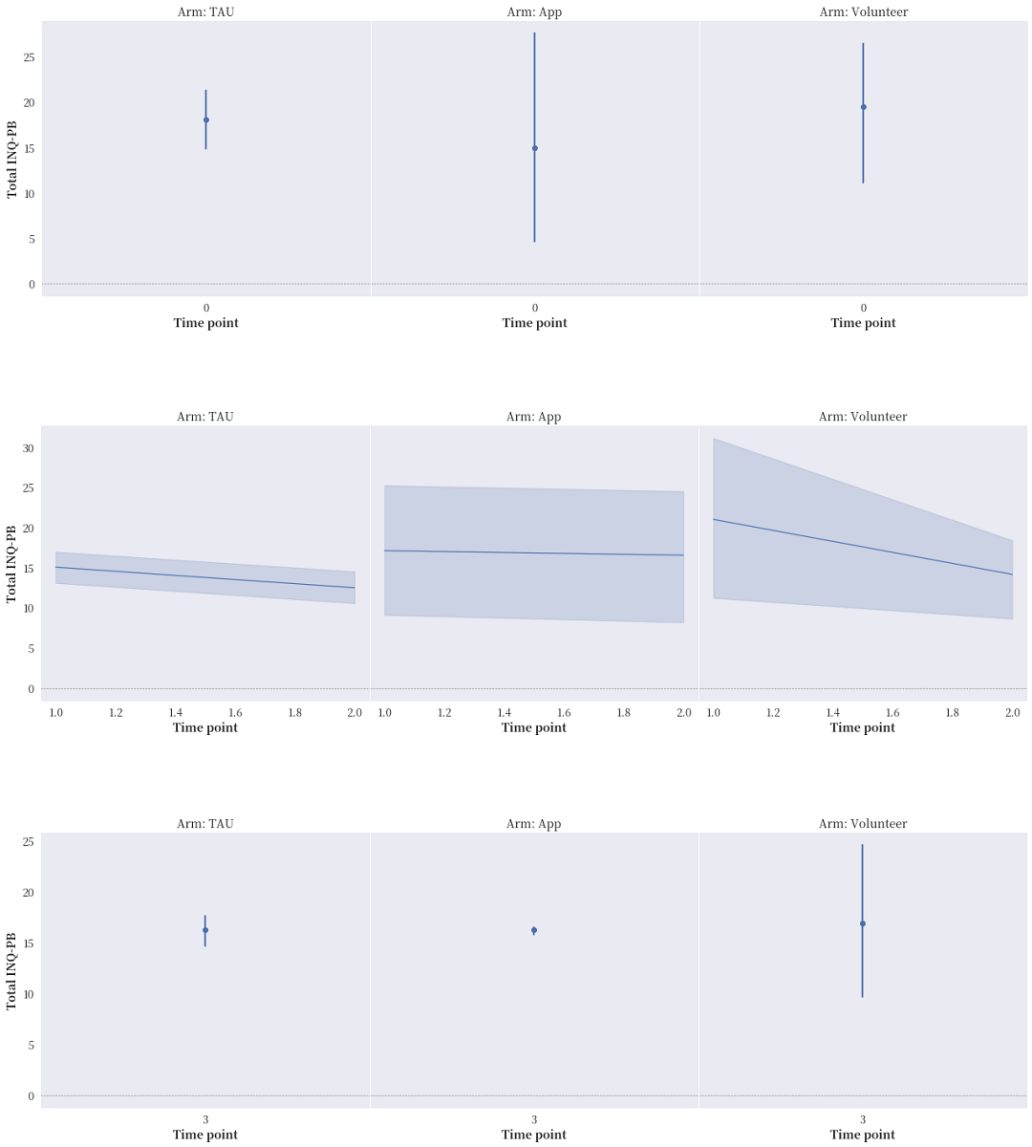
The effects of the intervention on Interpersonal Needs Questionnaire-Perceived Burdensomeness items (INQ-PB) at the 4 time points with intention-to-treat (ITT) analysis applied. TAU: treatment as usual.

**Figure 5:**
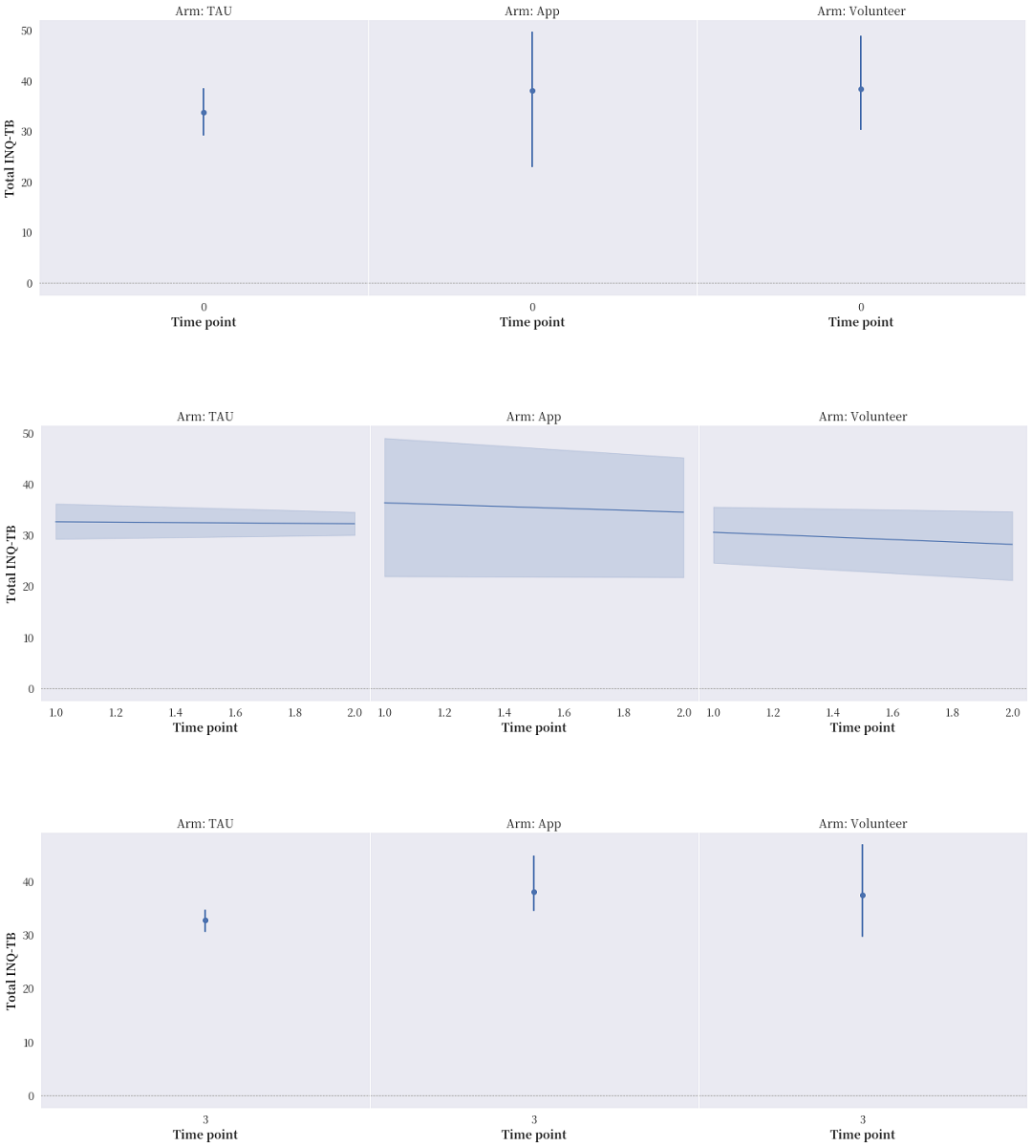
The effects of the intervention on Interpersonal Needs Questionnaire-Thwarted Belongingness items (INQ-TB) at the 4 time points with intention-to-treat (ITT) analysis applied. TAU: treatment as usual.

The proportions of reported compliance with service treatment and the seeking of professional and informal help in the “app” and “volunteer” groups were much higher in general than that in the “TAU” group. After applying ITT (Figures 2-5), “app” only led to better improvement in hopelessness than either “TAU” or “volunteer” at time points T1 and T2, but it brought no noticeable improvement to ASIQ4 and INQ. However, between the same time interval, “volunteer” was effective in bringing down ASIQ4, BHS4, and INQ at rates equal to or better than “TAU.” Much of the change in “volunteer” INQ was due to a noticeable fall in PB scores and a gradual decline in TB scores relative to “app” and “TAU.” A larger sample size is needed to determine the effective size and ascertain the mediation effects in the interventions (ie, to verify the existence of causal pathway TB>BHS4>ASIQ4).

## Discussion

### Principal Findings

The idea behind mixing volunteer care and a preprogrammed mobile app to engage discharged patients at the community level was to strengthen the continuity of care as a strategy to alleviate suicide risk through social connectedness and self-assurance, 2 categories of interpersonal needs. This contact was also expected to help them keep their standard medical follow-ups. The interventions were designed to address the aforementioned 2 categories of interpersonal needs with the aim of reducing patients’ sense of hopelessness as a predictor of suicidal ideation. The participating volunteers were stringently selected and trained to respond spontaneously based on human nature. The care scheme was piloted and tested [16] prior to the study, and the mobile app was preprogrammed to offer automated feedback to those in distress. Both interventions were well-structured, trial-run, and offered in a highly disciplined and ethical manner. No participant reported readmission to the hospital because of self-harm.

After applying ITT analysis, several important preliminary findings are particularly worthy of note. First, over time, both the “volunteer” and control groups reported an obvious improvement in suicidal ideation, whereas the “app” group did not. The ASIQ4 scores of all 3 groups bounced back to preintervention levels at T3 after the intervention period ended. Second, the control group reported worsened service compliance over time, whereas both the “volunteer” and “app” groups displayed improvement in this outcome during the intervention period. This finding is consistent with these participants’ efforts to seek professional consultation and informal help. Third, during the intervention period, all 3 groups reported improvement in interpersonal need fulfillment, but the “volunteer” group reported a more obvious and specific reduction in PB, as well as a gradual decline in TB. Fourth, the “app” group performed much better on the BHS4 across all the time points than the 2 other groups did. However, the “volunteer” group performed the best on suicidal ideation. Finally, despite the limitation of not being able to perform mediation analysis, the mixed volunteer plus app intervention seemed to produce the chain of effects theoretically hypothesized: from a reduction in INQ to hopelessness and then possibly to suicidal ideation. As can be seen in Table 4, the correlations among all the measurement scales (ASIQ4, BHS4, INQ, and CESD) were statistically significant, with the exception of those between BHS4 and PB. However, BHS4 was correlated with TB and the overall INQ, suggesting that BHS4 had a stronger interdependence with TB than PB. Ma et al [31] reviewed 58 mainly cross-sectional studies testing the IPTS. PB was found to be more commonly tested, and more support was found for its effect on suicidal ideation than TB [31]. TB might play a unique role in this study, which concurs with the longitudinal survey of youths conducted by Roeder and Cole [32], who found that TB can predict both hopelessness and PB over time, whereas a combination of the 3 factors can predict suicidal ideation. In this study, the reductions in PB and TB were only achieved with volunteer involvement. The “volunteer” group also had improved hopelessness and suicidal ideation over the other 2 groups.

This study provides initial evidence supporting the safety and viability of an intervention that combines personalized care by volunteers with programmed care by a mobile app for engaging postdischarge young adults with self-harm behaviors on their pathway to care. The intervention offers promising effects in reducing feelings of hopelessness and suicide risk by addressing patients’ interpersonal need for belongingness, although these effects were not shown to be sustainable after intervention in this study. However, caring contact by nature is not meant to be therapeutic; rather, it is aimed at better engagement to help discharged patients weather the transition from clinical to community care [4].

More evidence on the use of apps to treat people with mental health issues has emerged in recent years, but it is not conclusive. For example, the use of an app as part of clinical treatment for suicide prevention was unexpectedly found to exert negative effects on suicide risk reduction in an adult clinical sample with mild-to-moderate symptoms of anxiety, depression, and adjustment disorders [33]. However, another app (LifeBuoy) with therapeutic elements was recently shown to reduce suicidal ideation to a moderate degree in a much larger community sample of young adults (N=455) [34].

In the “app” arm of this study, the preprogrammed mobile app itself (ie, without caring contact by human volunteers) provided distinct value in alleviating the sense of hopelessness among the participants, who belong to the digital generation. The app, albeit basic, offers a clear buffer to help people in distress feel a sense of connection and feel less desperate. This study thus validates the role of a mobile app with basic self-help features in alleviating a sense of loneliness and hopelessness, both of which are strong predictors of suicide risk [35].

The smartphone penetration rate in Hong Kong rose from 92.1% in 2020 to 92.9% in 2021 [22], with the rise particularly notable during the COVID-19 pandemic. Mobile apps have become part of people’s decision-making and problem-solving processes. For example, they were widely used for information-sharing, risk assessment, training, and self-management during the pandemic [36]. With such a wide reach and the ability to be offered at a very low cost, digital support seems to be a viable auxiliary for individuals at risk of suicide when standardized medical follow-up care is made available as a necessary component.

It must be noted, however, that even though the “app” group realized a clear reduction in hopelessness, it showed no improvement in suicidal ideation, whereas the “volunteer” group reported improvements across the whole spectrum (BHS4, ASIQ4, and INQ). These results suggest that care rendered by trained volunteers remains critical in leading to the desired outcome change. More importantly, the volunteers were found to cause no additional harm, which aligns with prior research on the effects of mental support offered by trained volunteers [16,37-40].

Volunteer support appeared to address interpersonal needs shortly after the intervention began, with a modest effect on TB at T1, which was 1 month after discharge from an emergency department. However, the alleviation of suicidal ideation and PB was not noticed until 2 months later at T2. Unlike a preprogrammed mobile app, human care requires sufficient time to build rapport, which is based primarily on mutual understanding and conducive feedback. It motivates people through warmth and spontaneity [19]. In this study, the blending of the 2 components worked very well, showing that they complement each other. Nevertheless, the bounce-back effect was obvious, suggesting that this type of caring contact is transient and reversible.

The control group reported steady improvements in suicidal ideation and depressive symptoms over time during the intervention period. Standard medical care at the emergency departments and medical follow-up as TAU ameliorated such symptoms and suicide risk substantially [40]. Standard care proved to benefit those patients at acute risk of suicide. Proper psychiatric assessments and medication prescriptions offered in a timely fashion and at regular intervals to patients with a suicide risk remain valuable and are necessary components of any intervention for people with self-harm with or without suicidal ideation.

### Limitations

The social unrest in 2019 and the COVID-19 pandemic from 2020 onward took a heavy toll on this study, particularly given that the sole recruitment sites were hospitals. Although we adopted several measures to increase the case admission rate, including briefings with medical staff, the multiple outbreaks of COVID-19 between 2020 and 2021 resulted in the full suspension of research at hospital sites. This study’s sample size was thus suboptimal, limiting the external validity of its findings. The volunteer support intervention was also unexpectedly interrupted from time to time. Face-to-face contact was difficult to render during the aforementioned periods. Nevertheless, all the volunteers involved in the study offered their unfailing support to the participants and exercised incredible patience and care.

We are also aware of the fact that the length of the self-administered questionnaire could be a bit overwhelming to our participants, which could bias the results from one way to another. The outcomes could also have been impacted by the unblinded nature of this study. As a cross-check of the major psychometric items was in line with the results reported by the instrument authors, we could only assume the rest of their responses were also genuine.

### Conclusions

The RCT reported in this paper suggested that a reduction in PB and TB through community-based caring contact, as a form of engagement, is possibly linked to improvements in hopelessness and suicide risk. In comparison with TAU, PB and TB played a role as an alleviating effect, albeit a transient one, in reducing the level of hopelessness, which is a strong predictor of suicide risk. This finding, as hypothesized by the IPTS as a theory of change, suggests that suicide risk can be improved by addressing the need for social connectedness, which is strongly correlated with hopelessness and suicidal ideation.

This study has provided preliminary evidence that illustrates the effectiveness of blending volunteer care with a preprogrammed mobile app as a strategy for strengthening the continuity of care to alleviate the risk of suicide. The alleviation of suicidal ideation cannot be achieved solely through computer-mediated support, with meaningful human interaction confirmed to exert a prospective effect on reducing such ideation over time. The combination of volunteer care with a mobile app should be explored in a larger study. When cautiously implemented, it will help discharged patients with self-harm transition from a hospital to a community setting.

## Appendix

Multimedia Appendix 1 CONSORT-eHEALTH checklist (V 1.6.1).

## Abbreviations

ASIQ: Adult Suicidal Ideation Questionnaire
ASIQ4: 4-item short-form Adult Suicidal Ideation Questionnaire
BHS: Beck Hopelessness Scale
BHS4: 4-item short-form Beck Hopelessness Scale
CESD: Center for Epidemiological Studies Depression Scale
DSM IV-TR: Diagnostic and Statistical Manual of Mental Disorders, 4th Edition, Text Revision
ICD-10: International Classification of Disease, 10th revision
ICH-GCP: International Conference on Harmonization of Technical Requirements for Pharmaceuticals for Human Use Guideline for Good Clinical Practice
INQ: Interpersonal Needs Questionnaire
INQ-PB: Interpersonal Needs Questionnaire-Perceived Burdensomeness items
INQ-TB: Interpersonal Needs Questionnaire-Thwarted Belongingness items
IPTS: interpersonal-psychological theory of suicide
ITT: intention to treat
PB: perceived burdensomeness
RCT: randomized controlled trial
TAU: treatment as usual
TB: thwarted belongingness

## Data Availability

The data sets generated and/or analyzed during this study are not publicly available due to the limitations set by the University of Hong Kong Human Research Ethics Committee, but they are available from the corresponding author on reasonable request. The submission of a Data Access Request Form is required.
